# Does the contralateral testicular volume decide the need for diagnostic laparoscopy in cases of unilateral impalpable undescended testis?

**DOI:** 10.1186/s12894-024-01455-2

**Published:** 2024-03-26

**Authors:** Ahmed Elrouby, Mahmoud Ghalab, Mostafa Kotb

**Affiliations:** 1 Department of Pediatric Surgery, Faculty of Medicine, Alexandria University, Alexandria, Egypt; 2 Department of Radiology, Faculty of Medicine, Kafrelsheikh University, Kafrelsheikh, Egypt

**Keywords:** Impalpable testis, Volume, Ultrasound

## Abstract

**Background:**

This study aimed the evaluation of the value of the calculated volume of a normal testis to predict the status of its contralateral impalpable side and hence decide the importance of laparoscopic exploration.

**Methods:**

Patients with unilateral impalpable undescended testis – as confirmed by clinical and sonographic examination- were enrolled in our prospective interventional study between November 2018 and August 2022 at Elshatby University Hospital, Faculty of Medicine, Alexandria University. The volume and three-dimensional diameter of the normal contralateral testis were measured by the pre-operative US using the formula: Volume = L x W x H x π/6, where L is the length, W is the width, H is the height, and was correlated with the intra-operative laparoscopic findings.

**Results:**

Seventy-six patients were included in our study. The age of the studied patients ranged between 6 months and 4 years with a mean of 2.17 ± 1.30 years; most of them were between one and three years old. Forty-six patients (60.5%) have left-sided impalpable testis and 30 patients (39.5%) have right-sided impalpable testis. The calculated volume of the contralateral normal testis was significantly larger in those patients who had both blind ending vas and vessels (0.89 ± 0.16) and in those who had an atrophic testis passing through the deep inguinal ring (DIR) –which was excised through the inguinal region- (0.83 ± 0.20) than in those patients who had their testes intra-abdominal (0.53 ± 0.18) or passing through the DIR to the inguinal region (0.80 ± 0.19). (Kruskal Wallis test; *p* < 0.001*).

**Conclusions:**

The calculated sonographic volume of a normal testis can predict the status of its contralateral impalpable side significantly with sensitivity & specificity of 75.0% & 88.89% respectively and a cut-off point of ≤ 0.674; hence, helps in parent counselling preoperatively.

**Trial registration:**

Name of the registry: Clinicaltrials.gov PRS. Trial registration number: NCT05933811. Date of registration: 10-7-2023 (retrospectively registered). URL of trial registry record: https://clinicaltrials.gov.

## Background

Undescended testis or cryptorchidism is considered the most common congenital genito-urinary abnormality faced in male neonates with an incidence of 3% in full-term babies and increasing up to 30% in preterm males. However, this incidence declines to about 1% by the age of 3 months as it descends to its normal position in about 80% of cases [1, 2]. Only 20% of undescended testis is impalpable being either vanishing due to an intra-uterine accident, agenetic, intra-abdominal, or inguinal with different degrees of dysplasia and atrophy [3].

The gold standard method for a definitive diagnosis and management of impalpable undescended testis is laparoscopy and according to its findings; the procedure is planned whether nothing in case of vanishing or agenetic testis, inguinal exploration in case the vas and vessels are found passing through the deep inguinal ring (DIR) or traction, Fowler-Stevens (FS) or one stage laparoscopic assisted orchiopexy in case of the intra-abdominal testis [3, 4].

The size of the contralateral testis has been used to predict the condition of its contralateral nonpalpable one while being explored by laparoscopy by many researchers [5]. Ultrasound (US) is considered by many surgeons as being an accurate, reproducible, and objective in situ tool for the assessment of testicular dimensions and volume and in many studies, it can guide the initial surgical approach [6]. However, the work aimed to assess the accuracy of the three-dimensional size as well as the volume of a normal testis as measured by ultrasonography in predicting the status of its contralateral nonpalpable counterpart; hence, helping in parent counselling preoperatively.

## Methods

Our prospective interventional study included children with unilateral nonpalpable undescended testis aged between 6 months to 4 years who presented to our institute from November 2018 to August 2022. Patients with bilateral nonpalpable undescended testis, those who underwent hormonal treatment and cases subjected to 1st stage orchidopexy were excluded from our study.

**Fig. 1:**
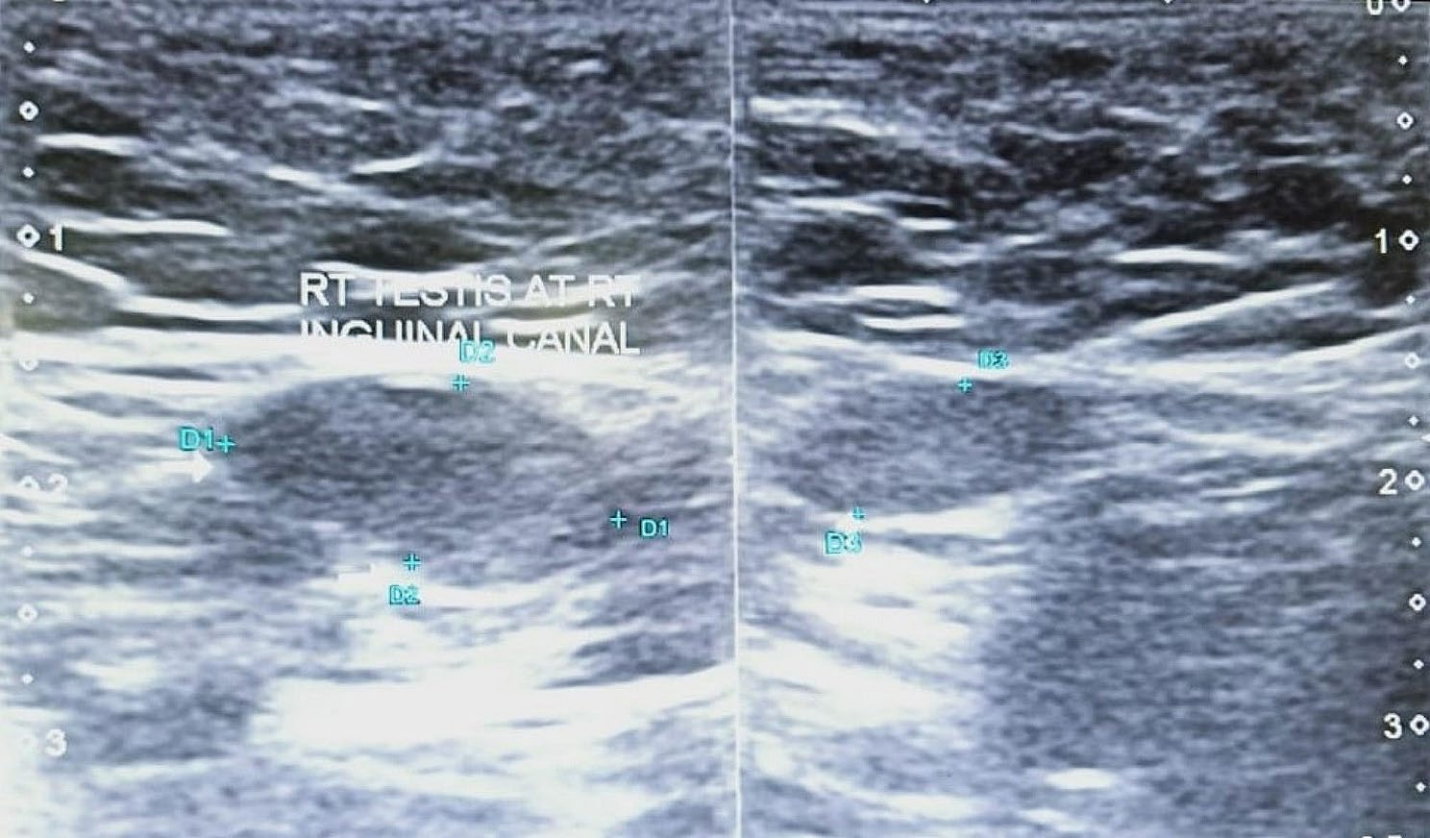
Measuring the dimensions of the testis using ultrasound. An US illustration showing the three dimensional measurement of a right testis at the inguinal region

After approval of the ethics committee of Alexandria Faculty of Medicine, informed consent was obtained from all parents and legal guardians of the children included in the study. Careful collection of the demographic data of all of the studied patients was followed by careful clinical examination of both hemiscrotum, inguinal regions and ectopic sites. US abdomen, pelvis, and the inguinoscrotal region performed by the same sonographer were done for:

1. Assessment of the three-dimension diameters as well as the volume of the normal-sided testis. It is measured by the formula: Volume = L x W x H x π/6, where L is the length, W is the width, and H is the height (Fig. 1). The volume of the contralateral (scrotal) testis was measured and reported. It is considered hypertrophied whenever its volume exceeds the reference values reported by Goede et al. [7]. [Figure 1]
2. Searching for the impalpable testis at all its suspected sites; intra-abdominal, pelvic, or at the inguinoscrotal region.

All of the studied patients were subjected to laparoscopic exploration under general anesthesia with endotracheal intubation and the following data regarding the findings of the affected impalpable side was collected:

A. Blind ending vas and vessels.
B. Intra-abdominal testis.
C. Cord structures passing through a closed DIR.
D. Cord structures passing through an open DIR.

The procedure which was done according to the laparoscopic findings was also documented.

A. Blind ending vas and vessels: Nothing.
B. Intra-abdominal testis: Traction 1, Fowler Stephens one or laparoscopic assisted orchiopexy.
C. Cord structures passing through the DIR (open or closed): Inguinal exploration and orchiopexy or excision in case of atrophied testis.

A contralateral testicular volume that is greater than the cutoff value was considered to be a positive predictive factor for monorchism. The test was considered as a true-positive result should the testis be absent during laparoscopic exploration. On the other hand, if a viable testis was found, the test was considered as a false-positive result. Sensitivity, specificity, and accuracy were calculated as well using these data.

### Statistical analysis

Data were fed to the computer and analyzed using IBM SPSS software package version 20.0. **(**Armonk, NY: IBM Corp**).** The **Shapiro-Wilk** was used to verify the normality of the distribution of variables, Comparisons between groups for non-parametric variables were assessed using the **Mann Whitney test** or **Kruskal Wallis test.** The significance of the obtained results was judged at the 5% level.

## Results

Our study included 76 male patients confirmed to have unilateral nonpalpable undescended testis as confirmed by clinical and sonographic examination. Their age ranged between 6 months and 4 years with a mean of 2.17 ± 1.30 years; most of them were between two and four years old. Forty-six patients had a left-side impalpable testis and 30 patients had a right-side impalpable testis **[**Table 1**].**

**Table 1:** Presentation of patients according to their age and the affected side (*n* = 76)

Parameter	N (%)
**Age (years)**	
< 1	23 (30.1%)
1–2	19 (25%)
2–4	34 (44.9%)
Mean ± SD.	2.17 ± 1.30
Median (Min. – Max.)	2.4 (0.5–4)
**Affected side**	
Right	30 (39.5%)
Left	46 (60.5%)

The median of the estimated testicular volume of the normal-sided testis among our studied patients was 0.74 ranging between 0.24 and 1.33 with an average of 0.72 ± 0.24. This volume was slightly lower in patients with affected on their right side than in patients with affected left side without showing statistical significance [Table 2: *p* = 0.230].

**Table 2:** The calculated volume of normal contralateral testis among the studied patients

Side of the affected impalpable testis	N	Volume of the contralateral normal testis			
		Mean ± SD.	Median (Min. – Max.)	Test of significance	p
**Right**	30	0.69 ± 0.25	0.67 (0.24–1.33)		
**Left**	46	0.75 ± 0.24	0.76 (0.26–1.19)	U = 577.0	0.230

**H**: Kruskal Wallis test, **U**: Mann Whitney test, **p**: p-value for comparing the studied categories, *: Statistically significant at *p* ≤ 0.05, **SD**: Standard deviation

Laparoscopic exploration of the impalpable undescended testis revealed variable findings among our studied patients. The affected testis was found intra-abdominal in 31 patients (40.79%) and the testicular vessel as well as the vas deferens were found passing through the deep inguinal ring (DIR) in 19 patients (25%). However, a blind-ending vas and vessels were found in one-third of our patients (26 patients) indicating a vanishing testis on this side. The highest calculated volume of the contralateral normal testis was measured in those patients who were found to have a blind-ending vas and vessels (0.89 ± 0.16) followed by patients with a passing vas and vessels through the DIR (0.82 ± 0.20) with the least calculated volume in patients who have an intra-abdominal testis (0.53 ± 0.18). The difference in the calculated volume of the contralateral normal testis in correlation with the intra-operative laparoscopic findings of the affected side was statistically significant as the volume of the normal contralateral testis in the case of the finding of intra-abdominal testis was significantly smaller than the measured volume of contralateral testis in the case of other laparoscopic findings. **[**Table 3: *p* < 0.001*].

The status of the DIR on the affected side was examined during laparoscopy and revealed that it was closed in 52 patients (68.42%) and patent in the remaining 24 patients (31.58%). The calculated volume of the contralateral normal side was larger in patients with closed DIR 0.81 ± 0.22 than in patients with patent rings (0.54 ± 0.17); this was statistically significant [Table 3: *p* < 0.001*].

**Table 3:** The operative findings of the studied patients (*n* = 76)

		Volume of the contralateral normal testis			
**The laparoscopic finding of the affected side**	**N (%)**	**Mean ± SD**	**Median (Min. – Max.)**	**Test of significance**	***P***
**Blind ending vas & Vessels**	26 **(**34.21%)	0.89 ± 0.16	0.86 (0.61–1.33)	H = 36.873*	< 0.001^*^
**Intra-abdominal testis**	31 (40.79%)	0.53 ± 0.18	0.53 (0.24–0.98)		
**vas & vessels passing through the DIR**	19 (25%)	0.82 ± 0.20	0.80 (0.50–1.19)		
**DIR of the affected side**	**N (%)**	**Mean ± SD.**	**Median (Min. – Max.)**	**Test of significance**	**p**
**Open**	24 (31.58%)	0.54 ± 0.17	0.54 (0.24–0.98)	U = 203.50*	< 0.001*
**Closed**	52 (68.42%)	0.81 ± 0.22	0.83 (0.30–1.33)		

**H**: Kruskal Wallis test, **U**: Mann Whitney test, **p**: p-value for comparing the studied categories, *: Statistically significant at *p* ≤ 0.05, **SD**: Standard deviation

The intra-operative procedure was done according to the laparoscopic findings. No further procedure was done in patients with a blind-ending vas and vessels (26; 34.21%). Patients with their vas and vessels passing through the DIR (19 patients) were subjected to inguinal exploration with inguinal orchiopexy in 9 patients (11.84%); all of them have an open DIR and excision of an atrophic remnant in the remaining 10 patients (13.15%); all of them have a closed DIR. Patients who have their testes intra-abdominal have different procedures according to their testicular status as shown in Table 4. The relation between the procedure done for the affected testis with the pre-operative calculated volume of the contralateral normal one was statistically significant (Table 4: *p* < 0.001*) where patients with the highest calculated volume having nothing done for their impalpable testis as they already have blind ending vas and vessels [Table 4**].**

**Table 4:** Surgical procedure for the affected side

	Volume of the contralateral normal testis				
Procedure is done for the affected side	N (%)	Mean ± SD.	Median (Min. – Max.)	Test of significance	p
**Nothing**	**26 (**34.21%)	0.89 ± 0.16	0.86 (0.61–1.33)	H = 37.521*	< 0.001^*^
**Traction1**	**17** **(22.36%)**	0.63 ± 0.23	0.61 (0.24–1.12)		
**Lap Assisted orchiopexy**	**12** **(15.78%)**	0.47 ± 0.12	0.50 (0.26–0.66)		
**Fowler Stephens one**	**2** **(2.63%)**	0.34 ± 0.11	0.34 (0.26–0.41)		
**Inguinal orchiopexy**	**9 (**11.84%)	0.80 ± 0.19	0.82 (0.57–0.99)		
**Atrophic & excised**	**10** **(13.15%)**	0.83 ± 0.20	0.79 (0.57–1.19)		
	**Volume of the contralateral normal testis**				
**The overall finding of** **the affected testis**	**N (%)**	**Mean ± SD.**	**Median (Min. – Max.)**	**Test of significance**	**p**
Absent	**36**	0.87 ± 0.17	0.84 (0.57–1.33)	U = 233.50*	< 0.001^*^
Present	**40**	0.59 ± 0.22	0.55 (0.24–1.12)		

**H**: Kruskal Wallis test, **p**: p-value for comparing between the studied categories, *: Statistically significant at *p* ≤ 0.05, **SD**: Standard deviation

The overall net result was that the impalpable testis was found and a certain interventional step for orchiopexy was done in 40 patients (52.63%) and it was absent in the form of being either not found (blind ending vas and vessels) or found atrophied and excised in the remaining 36 patients (47.37%). The calculated volume of the contralateral testis was significantly higher in those patients with a net result of an absent testis on the affected side. [Table:4, *p* < 0.001*]

**Fig. 2:**
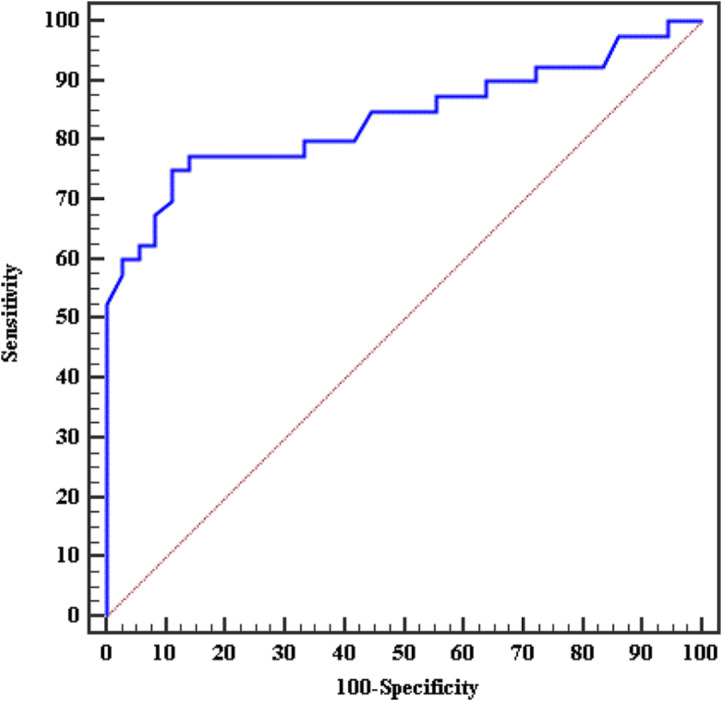
ROC curve for the volume of normal side to predict the presence of a second testis

The measured sensitivity & specificity of the importance of the calculated volume of the normal testis in predicting the status of the contralateral impalpable testis were 75.0% & 88.89% respectively. The overall result was that the calculated volume of the normal contralateral testis can significantly predict the status of the affected impalpable testis with a cutoff point of ≤ 0.674 cm. [Figure 2; Table 5: *P* < 0.001*]

**Table 5:** Validity (AUC, sensitivity, specificity) for the volume of normal side to predict the presence of the second testis

	AUC	P	95% C.I	Cut off	Sensitivity	Specificity	PPV	NPV
**Volume of the normal side**	0.838^*^	< 0.001^*^	0.744–0.931	≤ 0.674	75.0	88.89	88.2	76.2

**AUC**: Area Under a Curve, **p-value**: Probability value, **CI**: Confidence Intervals, **NPV**: Negative predictive value, **PPV**: Positive predictive value, *: Statistically significant at *p* ≤ 0.05, **# Cut-off** was chosen according to the Youden index

## Discussion

The size of the contralateral testis has been used by many authors in predicting the condition of its contralateral nonpalpable counterpart [5, 8]. Testicular hypertrophy in cases of absent or small-sized contralateral testis was first described by Laron and Zilka in 1969 [9]. Testicular volumes can be measured using different types of orchidometers or calipers. Prader’s orchidometer is the most widely used; nevertheless, US measurements of testicular volume are highly accurate, reproducible, and considered by many authors the best tool for measuring the testicular volume [10].

Seventy-six patients were included in our study in two consecutive years with an average age ranging between 6 months and 4 years in comparison to a study conducted by Shalaby et al. who included only 40 patients within 2 years with an age ranging between one and 6 years old [11]. The testicular volume in that study ranged from 0.94 to 6.33, with a mean of 3.12 ± 1.41 cm^3^. The difference compared to ours may be attributed to higher age groups being included in that study.

The result of laparoscopic exploration revealed 47% with absent testis and 53% with their testis present. This is approximately similar to the findings in other related studies as shown by AbdElsalam et al. who found the testis in about 57.5% of their studied patients and an absent testis in the remaining 42.5% [12].

The testicular volume as measured by US was significantly higher in those patients who had been found to have an absent testis (blind ending vas and vessels) or an excised atrophied canalicular remnant than in those who had a testis either intra-abdominal or passing into the inguinal canal. This confirms the concept of the importance of testicular hypertrophy in predicting the condition of its contralateral one as concluded also by Boehm et al. in their study [5].

The cut-off point of volume of normal testis in our study was ≤ 0.674 cm; this volume differs greatly from other studies as concluded by Hodhod et al. in their study in which the cut-off point was > 2 ml. However, in this study, the authors used Takihara orchidometer for measuring the testicular volume [13]. Although this orchidometer is useful in comparing different testicular sizes; nevertheless, the absolute values obtained are overestimated in many clinical conditions [14].

The estimated sonographic volume of the normal testis (cut-off point ≥ 0.674 cm) cm predicted significantly the condition of its contralateral impalpable one in our study. This is supported by the findings of Y. Wei et al. cut-off point > 0.65 cm) who concluded in their study that this estimate can predict significantly monorchism. Despite their findings, they emphasized the importance of laparoscopic exploration [15].

The laparoscopic exploration revealed closed DIR on the impalpable side in about 68% of our studied patients with significantly larger estimated testicular volume in those with closed ring 0.81 ± 0.22 than in patients with patent ring 0.54 ± 0.17. Ueda et al. concluded in their study evaluating the importance of the condition of DIR in case of unilateral impalpable testis that a closed DIR denotes the presence of an extra-abdominal nubbin or even absent findings and hence inguinal exploration in such cases could be avoided [16]. On the other hand, a survey involving more than 400 surgeons, around 92% and 75% of respondents preferred to explore the inguinal canal in case of open and closed DIR, respectively. This is because they believe that intracanalicular atrophic testis carry the risk of developing testicular cancer in the future [17].

The sensitivity of preoperative estimated testicular size at a cut-off point ≥ 0.674 cm in our study was 75% in the prediction of absent or atrophic contralateral impalpable one. This accuracy rate is considered intermediate among other studies as a higher accuracy (90%) was concluded by Hurwitz et al. in their study at a cut-off point of > 1.8 cm [8]. On the other hand, a study conducted by Hodhod et al. declared a sensitivity of 71.7% at a cut point-off > 2 ml [13]. In a more recent systematic review, it was concluded that defining a cut point for confirming contralateral testicular hypertrophy cannot demonstrate the condition of monorchism [18].

Hurwitz et al. concluded in their study that despite contralateral testicular hypertrophy can predict an absent contralateral one -in patients presented with unilateral impalpable undescended testis- with an accuracy of 90%, they recommended exploratory laparoscopy as it is the only diagnostic tool which could be done in such cases [8].

## Conclusion

A hypertrophied testis with a cutoff point of ≥ 0.674 cm can predict the absence of a contralateral nonpalpable undescended testis and hence play an important role in counseling parents preoperatively. However, the confirmatory rule of diagnostic laparoscopy cannot be excluded.

## Data Availability

The datasets used and/or analyzed during the current study are available from the corresponding author upon reasonable request.
